# Effect of neuromuscular electrical stimulation combined with early rehabilitation therapy on mechanically ventilated patients: a prospective randomized controlled study

**DOI:** 10.1186/s12890-023-02481-w

**Published:** 2023-07-21

**Authors:** Ying Liu, Yangyang Gong, Chaofan Zhang, Pingping Meng, Yubiao Gai, Xiaoning Han, Zhiyong Yuan, Jinyan Xing, Zehua Dong

**Affiliations:** 1 Department of Critical Care Medicine, The Affiliated Hospital of Qingdao University, No. 16, Jiangsu Road, Qingdao, Shandong 266000 China; 2 Department of Rehabilitation Medicine, The affiliated hospital of Qingdao University, Qingdao, Shandong 266000 China

**Keywords:** Mechanical ventilation, Neuromuscular electrical stimulation, Rehabilitation, Ultrasound

## Abstract

**Background:**

This study aimed to investigate the effectiveness of neuromuscular electrical stimulation (NMES) blended with early rehabilitation on the diaphragm and skeletal muscle in sufferers on mechanical ventilation (MV).

**Method:**

This is a prospective randomized controlled study. Eighty patients on MV for respiratory failure were divided into a study group (40 cases) and a control group (40 cases) randomly. The study group adopted a treatment method of NMES combined with early rehabilitation and the control group adopted the method of early rehabilitation only. The diaphragmatic excursion (DE), diaphragmatic thickening fraction (DTF), variation of thickness of intercostal muscles (TIM), variation of thickness of rectus abdominis (TRA), and variation of the cross-sectional area of rectus femoris (CSA-RF) were measured to evaluate the therapeutic effect by ultrasound before and after intervention at the first day of MV, the 3rd and 7th day of intervention and the day discharged from ICU.

**Results:**

No significant difference was found in the general demographic information and ultrasound indicators between the two groups before treatment (all P > 0.05). After treatment, the variation of DTF (0.15 ± 0.05% vs. 0.12 ± 0.04%, P = 0.034) was significantly higher in the study group than that in the control group on the day discharged from ICU. The variation of TRA (0.05 ± 0.09% vs. 0.10 ± 0.11%, P = 0.029) and variation of CSA-RF (0.13 ± 0.07% vs. 0.19 ± 0.08%, P < 0.001) in the study group were significantly lower than that in the control group. The duration of MV in the study group was significantly shorter than that in the control group [109.5 (88.0, 213.0) hours vs. 189.5 (131.5, 343.5) hours, P = 0.023]. The study group had better muscle strength score than the control group at discharge (52.20 ± 11.70 vs. 44.10 ± 15.70, P = 0.011).

**Conclusion:**

NMES combined with early rehabilitation therapy is beneficial in reducing muscle atrophy and improving muscle strength in mechanically ventilated patients. This treatment approach may provide a new option for patients to choose a rehabilitation program; however, more research is needed to fully evaluate the effectiveness of this treatment option.

**Supplementary Information:**

The online version contains supplementary material available at 10.1186/s12890-023-02481-w.

## Background

Mechanical ventilation (MV) is an effective way to treat patients with respiratory failure. However, the MV can also bring about complications such as ventilator-associated lung injury and ventilator-associated pneumonia (VAP) [1, 2]. In patients requiring MV, muscle atrophy and decreased muscle strength due to long-term bed immobilization, sepsis inflammatory factors, and the use of sedative drugs lead to offline difficulties and prolonged intensive care unit (ICU) stay [3]. In addition, patients with respiratory failure themselves have a variety of pathophysiological changes such as airway restriction, emphysema and gas retention. At the same time, adverse factors such as hypoxia, acidosis and oxygenation accumulation often occur in the body, which can lead to oxidative stress in the human body, resulting in internal pathological changes of the diaphragm, such as flattening and thinning of the diaphragm [4].

Over the years, notable efforts have been dedicated to defining the most superb strategy to weaning sufferers from MV. Early mobilization in the ICU is advisable to seriously sick sufferers by way of decreasing the incidence of ICU-acquired weakness, accelerating the functional restoration of patients, and accelerating liberation from the ventilator [5]. Comprehensive early rehabilitation has been proved to be effective in increasing awareness, reducing the incidence of complications, and shortening length of stay and MV in ICU patients [6]. Previous studies have shown that early rehabilitation can help ameliorate orate diaphragm dysfunction and get off the ventilator in MV patients [7].

Neuromuscular electrical stimulation (NMES) is not a new method and is widely used in stroke rehabilitation and exercise training [8, 9]. The significance of NMES in clinical application is mainly to improve the muscle strength of patients, increase the range of motion, reduce edema, reduce atrophy, reduce pain, and finally achieve the purpose of promoting tissue healing [10]. Other study have showed that the duration of invasive mechanical ventilation decrease with the use of NMES [11]. However, there are few prospective controlled studies to report the therapeutic effect of NMES combined with traditional rehabilitation methods on MV patients. The present study aimed to explore the treatment effects of NMES combined with early rehabilitation on patients with MV and to identify synergistic effects. It is hoped to provide theoretical basis for further weaning and reducing duration of MV.

## Methods

### Study population

**Fig. 1:**
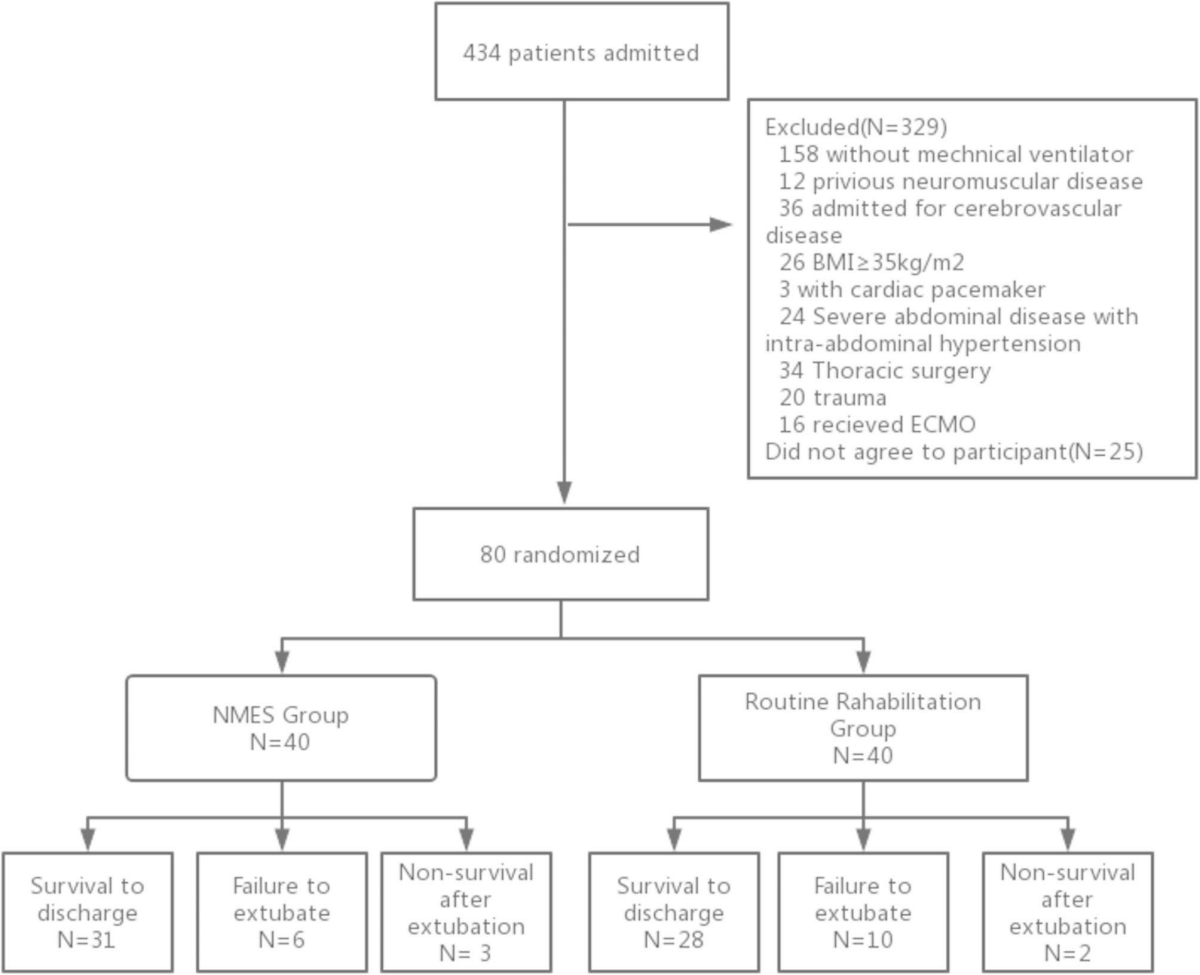
The weaning program All patients will be evaluated daily from 7:00am to 12:00am. Postoperative patients will be evaluated at any time according to their condition

This is a prospective randomized controlled study that conducted in the 32-bed medical ICU of a 2000-bed medical center in Affiliated Hospital of Qingdao University between February 2022 and September 2022. Patients on MV for respiratory failure were assigned to study group and control group randomly through a computer-generated allocation order. The study group adopted the method of NMES combined with early rehabilitation while those in the control group underwent regular rehabilitation without the application of NMES (Fig. 1).

The inclusion criteria were as follows: (1) Patients were expected to require MV > 72 h; (2) Patients and their relatives had written informed consent. The exclusion criteria:(1) Pregnancy; (2) Patients with preexisting neuromuscular lesions; (3) Patients with unrelieved pneumothorax and restricted diaphragm movement disorders; (4) Chest or diaphragm malformation; (5) Patients who cannot attach electrodes due to local skin damage or infection; (6) Patients with implanted pacemakers; (7) Patients that with a body mass index > 35 kg/m^2^; (8) Patients with severe intestinal gas accumulation, structural abnormalities, and other reasons that prevent ultrasound detection of diaphragm movement; (9) Patients already receiving palliative care and those with a survival expectancy of no more than 7 days.

**Fig. 2:**
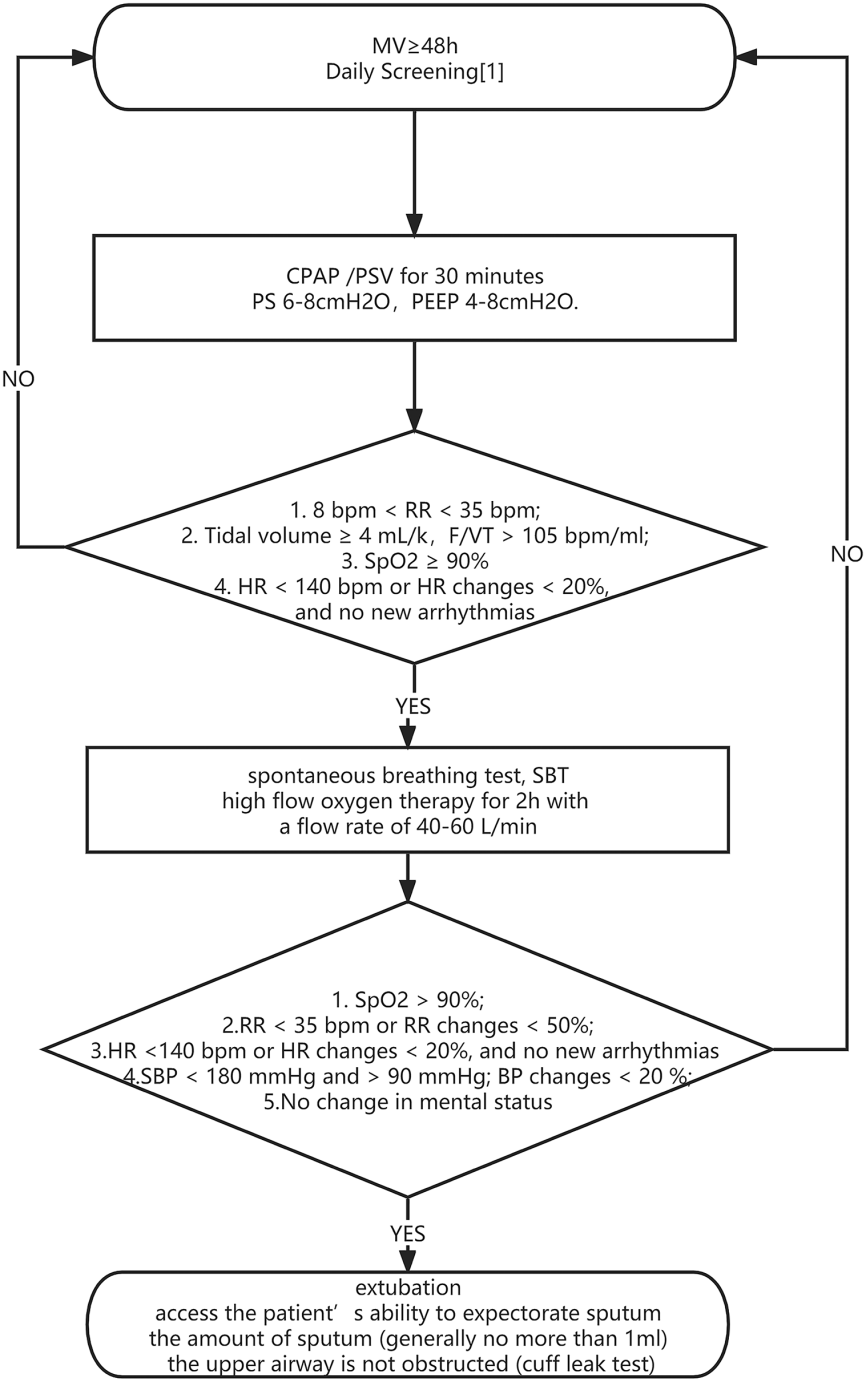
Flow diagram of the study

The ICU team rounds were made at least once a day, and doctors remain on duty 24 h a day. The ratio of patients to intensivists was 3:1 and the ratio of patients to nurses was 1:2. The ICU has a specialized rehabilitation technician, but not a respiratory therapist. The decision to remove endotracheal intubation was made by clinicians according to clinical experience and the weaning process, which was shown in Fig. 2.

This study was approved by the Ethics Committee of the Affiliated Hospital of Qingdao University (No.QYFYWZLL26720) and has been registered at clinicaltrials.gov. with trial registration number NCT05217511.

### Collection of clinical indicators

General clinical data obtained included the following: (1) Basic characteristics, including age, gender, Acute Physiology and Chronic Health Evaluation (APACHE) II scores [12] within 24 h of ICU admission, Body Mass Index (BMI),complications expressed as age-adjusted Charlson Comorbidity Index (aCCI) [13], causes for ventilator including pulmonary etiology and non-pulmonary etiology including acute respiratory distress syndrome caused by cardiogenic and abdominal diseases etc.; (2) Rehabilitation information, including frequency of neuromuscular electrical stimulation therapy and early rehabilitation therapy, occurrence of adverse events; (3) Treatment outcomes, including patient prognosis, duration of mechanical ventilation, duration of tracheal intubation, length of hospital stay and ICU stay and Medical Research Council (MRC) score at discharge. The MRC included an overall score ranged from 0 (total paralysis) to 60 (normal muscle strength) [14].

### Neuromuscular electrical stimulation therapy

**Fig. 3:**
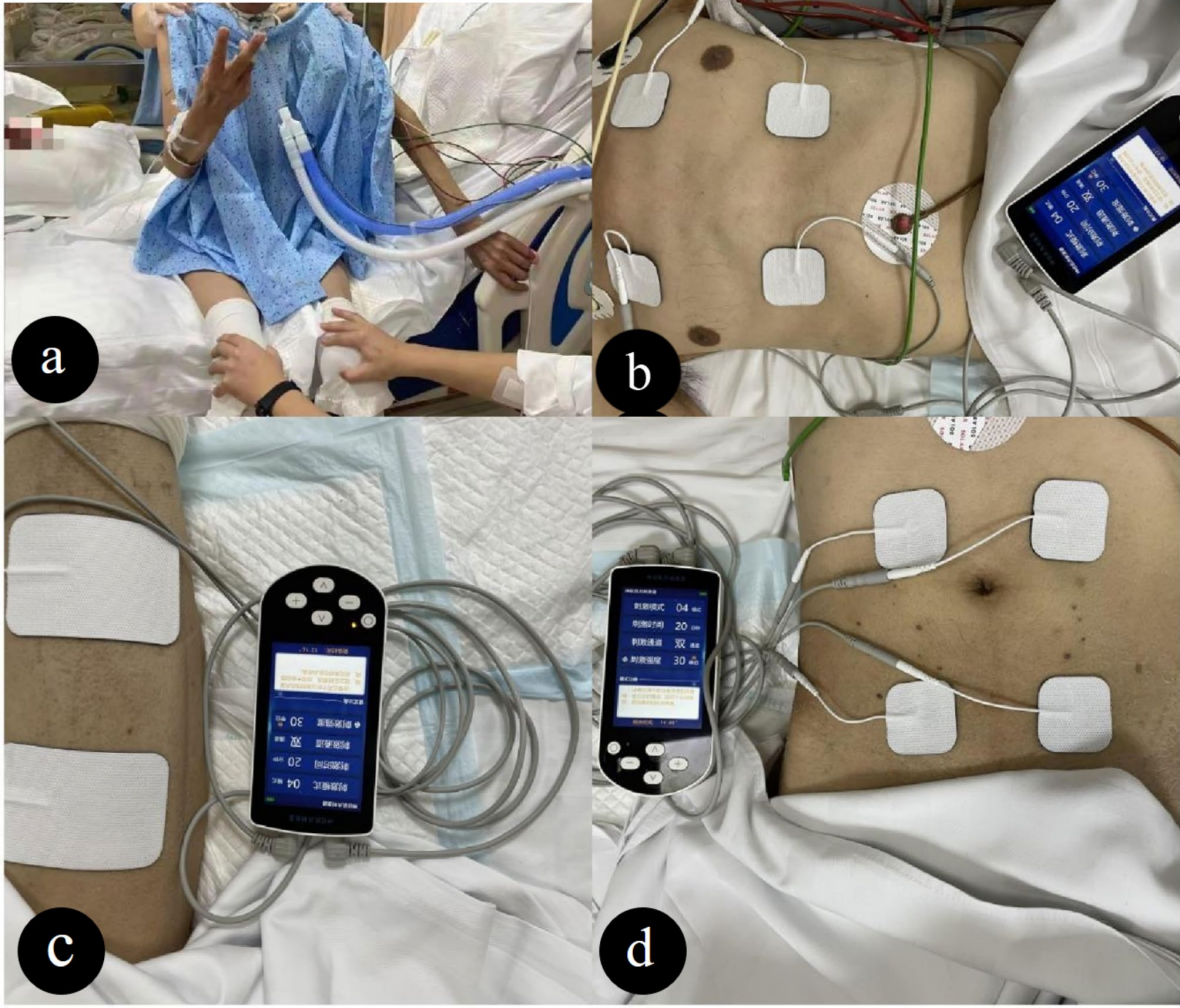
Neuromuscular electrical stimulation therapy. (**a**) early rehabilitation, the mechanically ventilated patients sit on the edge of the bed with the help of staff; (**b**) Intercostal muscle neuromuscular electrical stimulation, the electrode was applied to the intercostal space to stimulate the intercostal muscle group; (**c**) Neuromuscular electrical stimulation of the quadriceps femoris, the electrodes were applied to the proximal and distal thigh muscles to stimulate the quadriceps muscle; (**d**) Neuromuscular electrical stimulation of rectus abdominis, The electrodes were applied to the abdomen to stimulate the rectus abdominis muscle with the intensity required to achieve muscle contraction. Each stimulation lasted for 30 min

Patients in the study group received the NMES intervention 30 min per day. NMES was performed using a portable machine named ResPower Respiratory Neuromuscular Stimulator (Yaguo, China). Negative electrodes were placed at the motion points of the pectoralis major fibers, rectus abdominis and bilateral quadriceps muscles. The positive electrode was located far from the first electrode, near the muscle being stimulated. It consists of a channel in which each muscle has two electrodes (Fig. 3). Each NMES session took about 30 min. The parameters were set as follows: 50 Hz frequency, pulse duration 300 ms, rise time 1 s, stimulus time 3 s, decay time 1 s, and relaxation time 10 s. Gradually increase the intensity of electrical stimulation until significant muscle contractions occur. Patients were encouraged to tolerate as much stimulation as possible during electrical stimulation. Patients should be relaxed during NMES to avoid spontaneous quadriceps contractions and co-contraction of leg muscles.

### Early rehabilitation program

An individualized early rehabilitation program was designed based on the physiological and psychological conditions of the patients, with the participation of ICU doctors, nurses and rehabilitation therapist [7]. Rehabilitation therapy consisted of six levels: level 0, turning over once every 2 h for unconscious patients with unstable vital signs; level 1–2, in addition to turning over, maintaining joint range of motion to prevent muscle atrophy, and placing normal limb position for conscious patients who could sit up for at least 20 min, 3 times a day; level 3, similar to level 2, but sitting on the edge of the bed for patients who could perform upper-limb anti-gravity training; level 4, similar to level 3, but standing up or sitting in a chair for at least 20 min a day for patients who could perform lower-limb anti-gravity training; and level 5, patients actively moved from the bed and walked bedside. The outcome of rehabilitation was assessed by the ICU mobility Scale [15] (Supplemental Table 1). The two groups were treated with the same clinical management, ventilation strategy, MV break, tracheostomy and other treatment regiments according to the ICU protocol.

### Ultrasound measurement

An ultrasound doctor with more than 5 years of experience performed the Ultrasound (US) evaluation at the first day of MV and 3rd, 7rd day of intervention and the day discharged from ICU with a Mindray TE-5 machine (Shenzhen Mindray Bio-Medical Electronics Co. Ltd. Shenzen, China).

Diaphragm US: The diaphragm was evaluated by ultrasound in all patients [7]. Measures included diaphragm offset (DE), which was used to indicate the amplitude of diaphragm movement during the breathing cycle. Diaphragmatic thickening fraction (DTF) was used to indicate the different thickness of the diaphragm at the end of expiratory and inspiratory breath. End-inspiratory diaphragm thickness (DTei) and end-expiratory diaphragm thickness (DTee) refer to the maximum and minimum values of each respiratory cycle, respectively. The DTF was calculated by DTF = (DTei DTee) / DTee 100%. Record the values for 3 consecutive breathing cycles and take the average value as the final value. The diaphragmatic ultrasound was performed in a semi-supine position (bed slope 45) and a right anterior subcostal view was preferred. Measurement of the thickness of intercostal muscles (TIM): Still images of the 2nd and 3rd parastrasternal intercostal muscles were taken in the sagittal plane at end-tidal inspiration. Muscle thickness was measured between the outermost and inner echogenic layer of the muscle fascial borders [16]. The thickness of rectus abdominis (TRA) muscle over upper abdomen was measured in the supine position. The thickest muscle parallel to the boundary of the deep rectus abdominis fascia and the thickness of the superficial rectus abdominis fascia were also assessed [17]. Measurement of the cross-sectional area of rectus femoris (CSA-RF): With the patient in the supine position, the lower limbs were naturally relaxed and straight. A probe with sufficient coupling agent was placed between the upper margin of the right lower limb patella and the anterior superior iliac spine. The probe was positioned to display the rectus femoris muscle and its cross-sectional area was delineated along the rectus femoris fascia [18]. A landmark was drawn on the skin with a permanent marker to ensure reproducibility across modalities and measurements. The results of US examination were compared with their initial status, and the muscle atrophy rate was calculated. Muscle atrophy rate = (Measurement results - D1 measurement results) / D1 measurement results.

### Statistical analysis

The statistical analysis was performed using the SPSS software program (version 22.0; IBM Corp, Chicago, IL, USA). Continuous variables satisfying normal distribution were represented by mean ± SD. Variables that do not meet the normal distribution are represented by the median (25-75% percentile range). The statistical tests involved in this study are all unpaired tests. The tests of significance were 2 tailed at an α error of 0.05. Baseline comparisons were performed using the chi-square test (to test for equal proportions) for categorical data, an independent t test for continuous normally distributed variables and the Wilcoxon rank-sum test for data that does not satisfy a normal distribution.

## Results

### Basic characteristics of patients

A total of 80 patients were finally enrolled in this study, with 40 patients in the study group and 40 patients in the control group. There was no significant difference in the general demographic information between the two groups (all P > 0.05) (Table 1).

**Table 1:** Basic Characteristics and Ultrasound measurements at basic status of patients

Index	Study group (n = 40)	Control group (n = 40)	P
Sex (male)			0.82
Male	25	23	
Female	15	17	
Age (year)	58.13 ± 15.54	59.08 ± 16.02	0.788
BMI (kg/m^2^)	24.82 ± 5.23	24.19 ± 4.13	0.553
Sepsis (n)	26	23	0.647
MOF (n)	17	19	0.822
Etiology			0.654
Pulmonary	23	20	
Non-pulmonary	17	20	
Charlson Comorbidity Index	4.50 ± 2.61	4.50 ± 2.47	1
Glucocorticoids (n)	4	5	1
Neuromuscular blockers (n)	4	8	0.348
Sedation			
midazolam	16	15	1
propofol	17	12	0.352
dexmedetomidine	12	16	0.482
Sedation (hours)	157 (97,210)	196 (136,343)	0.05
APACHE II Score	20.55 ± 6.40	19.78 ± 6.44	0.591
Nutrition Score (NRS-2002)	4.25 ± 1.50	4.50 ± 1.16	0.406
DE (cm)	1.34 ± 0.56	1.26 ± 0.45	0.502
DTF (%)	0.11 ± 0.04	0.12 ± 0.34	0.131
TIM (cm)	0.49 ± 0.12	0.48 ± 0.12	0.97
TRA (cm)	0.80 ± 0.20	0.75 ± 0.21	0.311
CSA-RF (cm^2^)	4.98 ± 1.34	4.82 ± 1.21	0.579

BMI, body mass index; APACHE II, Acute Physiology and Chronic Health Evaluation (II); DE, diaphragmatic excursion; DTF, diaphragmatic thickening fraction; TIM, thickness of intercostal muscles; TRA, thickness of rectus abdominis; CSA-RF, cross-sectional area of rectus femoris; MOF multi-organ failure

### Ultrasound measurements before rehabilitation

There were no significant differences between the study group and the control group in DE (1.34 ± 0.56 cm vs. 1.26 ± 0.45 cm, P = 0.502), variation of DTF (0.11 ± 0.04% vs. 0.12 ± 0.34%, P = 0.131), TIM (0.49 ± 0.12 cm vs. 0.48 ± 0.12 cm, P = 0.970), TRA (0.80 ± 0.20 cm vs. 0.75 ± 0.21 cm, P = 0.311), and CSA-RF (4.98 ± 1.34cm^2^ vs. 4.82 ± 1.21cm^2^, P = 0.579) at basic status (Table 1).

### Ultrasound measurements after rehabilitation

After rehabilitation protocol, the DE, DTF, TIM, TRA and CSA-RF showed no differences between the two groups at 3rd and 7th day of intervention. The DTF was significantly higher in the study group than that in the control group at the day discharged from ICU (0.15 ± 0.05% vs. 0.12 ± 0.04%, P = 0.034). The variation of TRA (0.05 ± 0.09% vs. 0.10 ± 0.11%, P = 0.029) and variation of CSA-RF (0.13 ± 0.07% vs. 0.19 ± 0.08%, P < 0.001) were also significantly lower in the study group than that in the control group at discharge from ICU (Table 2).

**Table 2:** Ultrasound measurements at the intervention stage

Index	Study group(n = 40)	Control group(n = 40)	P
DE (cm)			
The 3rd day of intervention	1.32 ± 0.33	1.38 ± 0.39	0.454
The 7th day of intervention	1.55 ± 0.36	1.45 ± 0.38	0.235
At discharged from ICU	1.52 ± 0.46	1.59 ± 0.40	0.465
DTF (%)			
The 3rd day of intervention	0.11 ± 0.05	0.10 ± 0.05	0.349
The 7th day of intervention	0.14 ± 0.06	0.13 ± 0.05	0.334
At discharged from ICU	0.15 ± 0.05	0.12 ± 0.04	0.034
TIM (cm)			
The 3rd day of intervention	0.46 ± 0.09	0.44 ± 0.12	0.491
The 7th day of intervention	0.45 ± 0.07	0.47 ± 0.11	0.27
At discharged from ICU	0.46 ± 0.07	0.44 ± 0.10	0.267
TRA (cm)			
The 3rd day of intervention	0.81 ± 0.19	0.73 ± 0.19	0.085
The 7th day of intervention	0.77 ± 0.18	0.72 ± 0.20	0.244
At discharged from ICU	0.76 ± 0.19	0.67 ± 0.17	0.038
CSA-RF (cm^2^)			
The 3rd day of intervention	4.76 ± 1.21	4.58 ± 1.14	0.508
The 7th day of intervention	4.50 ± 1.16	4.30 ± 1.11	0.431
At discharged from ICU	4.31 ± 1.11	3.88 ± 0.85	0.053
Variation of TIM (%)			
The 3rd day of intervention	0.03 ± 0.15	0.07 ± 0.05	0.222
The 7th day of intervention	0.05 ± 0.19	0.04 ± 0.27	0.815
At discharged from ICU	0.03 ± 0.19	0.08 ± 0.20	0.209
Variation of TRA (%)			
The 3rd day of intervention	0.03 ± 0.20	0.02 ± 0.12	0.198
The 7th day of intervention	0.02 ± 0.16	0.07 ± 0.22	0.323
At discharged from ICU	0.05 ± 0.09	0.10 ± 0.11	0.029
Variation of CSA-RF (%)			
The 3rd day of intervention	0.03 ± 0.13	0.04 ± 0.16	0.915
The 7th day of intervention	0.07 ± 0.22	0.09 ± 0.11	0.349
At discharged from ICU	0.13 ± 0.07	0.19 ± 0.08	< 0.001

DE, diaphragmatic excursion; DTF, diaphragmatic thickening fraction; TIM, thickness of intercostal muscles; TRA, thickness of rectus abdominis; CSA-RF, cross-sectional area of rectus femoris

### Outcomes of patients

The duration of MV in the study group was significantly shorter than that in control group [109.5 (88.0, 213.0) hours vs. 189.5 (131.5, 343.5) hours, P = 0.023]. However, the duration of intubation showed no significant difference [159.5 (108.0, 231.0) hours vs. 209.0 (149.0, 374.5) hours, P = 0.100]. Patients in the study group had significantly higher muscle strength score than that in the control group at discharge (52.20 ± 11.70 vs. 44.10 ± 15.70, P = 0.011). Four patients in the study group suffered adverse effects, including increased blood pressure, decreased oxygen saturation, and intractable hiccups. One patient in the study group experienced a drop in blood pressure during routine rehabilitation and recovered after stopping rehabilitation (Table 3).

**Table 3:** Comparison of treatment results between the two groups

Results	Study group (n = 40)	Control group (n = 40)	P
Duration of MV (hour)	109.5(88.0,213.0)	189.5(131.5,343.5)	0.023
Duration of intubation (hour)	159.5(108.0,231.0)	209.0(149.0,374.5)	0.100
Length of Hospital Stay(day)	23.98 ± 13.95	25.08 ± 17.28	0.756
Length of ICU Stay (day)	17.85 ± 10.27	19.30 ± 14.96	0.615
IMS ≥ 3	14	9	0.323
MRS-Score (points)	52.20 ± 11.70	44.10 ± 15.70	0.011
Time of rehabilitation	11.14 ± 5.80	11.79 ± 9.32	0.719
Time of NMES	10.5 ± 5.96		
Number of adverse reactions	4	1	0.359
Treatment outcomes			0.509
Survival to discharge	31	28	
Failure to extubate	6	10	
Non-survival after extubation	3	2	

MV, Mechanical ventilation; ICU, intensive care unit; NMES, neuromuscular electrical stimulation; IMS, ICU Mobility Scale, MRC: Medical Research Council score

## Discussion

Our study found that NMES improves muscle strength in patients on MV and slows muscle atrophy in skeletal muscles such as rectus abtris and quadriceps femoris when combined with conventional rehabilitation therapy, and improves muscle strength at discharge.

Intensive rehabilitation refers to the rehabilitation treatment in intensive care environment, which is based on 24-hour close medical supervision and collaboration of multidisciplinary teams, with the purpose of preventing complications, preventing functional degradation and dysfunction, and improving functional activity ability and quality of life[19]. In severe patients, due to sedation, analgesia, mechanical ventilation, neuromuscular hysteresis agents and poor nutritional status, muscle atrophy and muscle strength decline is very obvious[20]. Therefore, it is important to use physical methods to maintain muscle strength in patients with MV. Leite et al.[21] found that for patients with non-neurological diseases, daily consecutive electrical stimulation sessions could shorten the duration of MV and improve the peripheral muscle strength and function, especially in patients with NMES of the quadriceps. Another study found that after 8 days of NMES, muscle loss in the quadriceps femoris and the peroneus longus was reduced, suggesting that NMES was beneficial for muscle retention [22].

Early pulmonary rehabilitation in patients with MV can be divided into passive exercise and active exercise. Early mobility and exercise, an important part of ABCDE bundle to prevent delirium in critical ill patients, has been proved feasible, safe, and beneficial for patients with prolonged MV in the ICU. Active mobilization and rehabilitation in the ICU improves mobility status, increases muscle strength, and elongates the survival time of patients.The combination of early mobility with other complementary care processes creates a holistic approach to clinical care, helps patients recover,and creates a philosophy of care that is greater than the sum of its parts [23].

Passive exercise is suitable for patients who cannot cooperate, including passive limb movement, passive bedside cycling, and NMES[24]. As an alternative therapy for mobilization and exercise, NMES is particularly suitable for patients who are bedridden. The involvement of motor units during NMES contraction differs from the underlying spontaneous activation. NMES activate sensory pathways through movement and cause depolarization of peripheral motor nerves[9]. However, the mechanism for strengthening muscle has not been fully understood. One theory is that NMES may increase muscle strength just like that of voluntary activity[25]. Another hypothesis is that NMES stimulate motor units to recruit type II fibers and cause contractions, which are the opposite of normal contractions, to achieve a more significant muscle-building effect than voluntary exercise alone[26].In addition, peripheral application of NMES can evoke a wide range of activities in the central nervous system, which can lead to a series of neural adjustments and adaptations[27].Therefore, the combination of NMES and routine rehabilitation may achieve the recruitment of more motor units, the recovery of nerve function, and the improvement of muscle strength. Previous study recommended that NMES should be performed for 60 min a day, with the recommended stimulation intensity of 45 Hz and the recommended frequency of once a day[28]. In this study, each NMES session lasted 30 min and the parameters were set as 50 Hz frequency. Four patients in the NMES group suffered adverse effects, including increased blood pressure and decreased oxygen saturation, and two of them suffered intractable hiccups, which resolved after discontinuation of neuromuscular electrical stimulation and medical treatment. One patient experienced a drop in blood pressure during routine rehabilitation and recovered after stopping rehabilitation. Although the rate and duration of NMES used in this study are different from those previously reported, the results of this study suggest that such frequency of operation is still safe and effective.

## Limitation

There still existed some limitations in this study. First, the present study was designed to be a single-center study with a small sample size. Multicenter trials with more les are still needed. Second, only the thickness and cross-sectional area of skeletal muscle were measured, and there may be measurement errors in patients with edema and local inflammation. At the same time, it should be emphasized that it is still difficult to accurately evaluate the influence of ventilation mode on diaphragm thickness change. Continuous positive airway pressure (CPAP) was adjusted whenever possible during ultrasound measurements to minimize the influence of breathing patterns on diaphragmatic measurements.

## Conclusion

NMES may be beneficial in slowing muscle atrophy and improving muscle strength at discharge in MV patients when combined with early rehabilitation therapy. Although this intervention may require prolonged use for benefits, it can be used as an alternative and supplement to routine rehabilitation.

## Electronic supplementary material

Below is the link to the electronic supplementary material.

Supplementary Material 1

## Data Availability

The data are available from the corresponding author on reasonable request.

## Abbreviations

NMES: neuromuscular electrical stimulation
MV: mechanical ventilation
DE: diaphragmatic excursion
DTF: diaphragmatic thickening fraction
TIM: thickness of intercostal muscles
TRA: thickness of rectus abdominis
CSA-RF: cross-sectional area of rectus femoris
VAP: ventilator-associated pneumonia
BMI: Body Mass Index
aCCI: age-adjusted Charlson Comorbidity Index
MRC: Medical Research Council
US: Ultrasound
DE: diaphragm offset
DTF: Diaphragmatic thickening fraction
DTei: End-inspiratory diaphragm thickness
DTee: end-expiratory diaphragm thickness
TRA: thickness of rectus abdominis
CSA-RF: cross-sectional area of rectus femoris
