# Supplementary material for: Effect of neuromuscular electrical stimulation combined with early rehabilitation therapy on mechanically ventilated patients: a prospective randomized controlled study
Source: BMC Pulm Med. 2023 Jul 21;23:272. doi: 10.1186/s12890-023-02481-w (PMC10362773; doi:10.1186/s12890-023-02481-w)
Supplement: Supplementary file 1 — Supplementary Material 1 [file 12890_2023_2481_MOESM1_ESM.docx]

| Classification | | Definition |
| --- | --- | --- |
| 0 | Nothing (lying in bed) | Passively rolled or passively exercised by staff, but not actively moving |
| 1 | Sitting in bed, exercises in bed | Any activity in bed, including rolling, bridging, active exercises, cycle ergometry and active assisted exercises; not moving out of bed or over the edge of the bed |
| 2 | Passively moved to chair (no standing) | Hoist, passive lift or slide transfer to the chair, with no standing or sitting on the edge of the bed |
| 3 | Sitting over edge of bed | May be assisted by staff, but involves actively sitting over the side of the bed with some trunk control |
| 4 | Standing | Standing Weight bearing through the feet in the standing position, with or without assistance. This may include use of a standing lifter device or tilt table |
| 5 | Transferring bed to chair | Able to step or shuffle through standing to the chair. This involves actively transferring weight from one leg to another to move to the chair. If the patient has been stood with the assistance of a medical device, they must step to the chair (not included if the patient is wheeled in a standing lifter device.) |
| 6 | Marching on spot (at bedside) | Able to walk on the spot by lifting alternate feet (must be able to step at least 4 times, twice on each foot), with or without assistance |
| 7 | Walking with assistance of 2 or more people | Walking away from the bed/chair by at least 5 metres (5 yards) assisted by 2 or more people |
| 8 | Walking with assistance of 1 person | Walking away from the bed/chair by at least 5 metres (5 yards) assisted by 1 person |
| 9 | Walking independently with a gait aid | Walking independently with a gait aid Walking away from the bed/chair by at least 5 metres (5 yards) with a gait aid, but no assistance from another person. In a wheelchair bound person, this activity level includes wheeling the chair independently 5 metres (5 years) away from the bed/chair |
| 10 | Walking independently without a gait aid | Walking away from the bed/chair by at least 5 metres (5 yards) without a gait aid or assistance from another person |

Supplemental Table 1：ICU mobility scale (IMS)
